# MRI metal artifact characterization in patients with spinal cord injury at 3 Tesla

**DOI:** 10.1038/s41393-026-01222-0

**Published:** 2026-05-29

**Authors:** Feroze B. Mohamed, Devon M. Middleton, Arichena Manmatharayan, James S. Harrop, Franz Kreidie, Joshua Fisher, Mahdi Alizadeh, Kiran Talekar, Ajit Karambelkar, Scott H. Faro, Adam E. Flanders, Laura Krisa

**Affiliations:** 1 Jefferson Integrated Magnetic Resonance Imaging Center, Department of Radiology, Thomas Jefferson University, Philadelphia, PA USA; 2 Department of Neurological Surgery, Thomas Jefferson University, Philadelphia, PA USA; 3 Department of Neurology, Thomas Jefferson University, Philadelphia, PA USA; 4 Department of Physical Therapy, Thomas Jefferson University, Philadelphia, PA USA; 5 Department of Occupational Therapy, Thomas Jefferson University, Philadelphia, PA USA; 6 Center for Outcomes and Measurement, Jefferson College of Rehabilitation Sciences, Thomas Jefferson University, Philadelphia, PA USA

**Keywords:** Musculoskeletal system, Diagnostic markers

## Abstract

**Study design:**

(Phantom & Human)

**Objective:**

The purpose of this study was to optimize imaging sequences to suppress artifacts induced by metallic hardware, using phantoms implanted with spinal hardware and participants with spinal cord injury (SCI) and spinal hardware at 3Tesla.

**Settings:**

US

**Methods:**

Magnetic resonance (MR) sequences were first tested on realistic agar-suspended spine phantom models with metallic instrumentation. C-spine with anterior-plate, posterior rods/screws, C-spine with rods/screws, C-spine with Kirschner wire, posterior T-spine with rods/screws, and anterior/posterior T-spine with anterior-plate and rods/screws were used. The optimized metal-suppression sequences obtained from phantom imaging were then evaluated on sixteen participants with SCI with similar metal implants. Four neuroradiologists performed a qualitative analysis and ranked all the scans, both with and without metal suppression. The following subjective visual assessment included: conspicuity of neural foramen, mitigation of artifact, visualization of the spinal cord and homogeneity of the cerebrospinal fluid (CSF).

**Results:**

Agreement between the raters was moderate (0.41 to 0.6) to substantial (0.61 to 0.8) for most measures, although some were in the fair range (0.21 to 0.4). In evaluating the T2 weighted-axial images for conspicuity of neural foramen, visualization of spinal cord, and homogeneity of CSF as well as T1 weighted-axial image for homogeneity of CSF in the anterior plate, the upper bound of the confidence interval was below “3” so the metal suppressed image was favored.

**Conclusion:**

There is some improvement in using metal-suppressed sequences to evaluate spinal cord injury patients with metal hardware at 3T MRI; however, the model-adjusted mean scores did not reach statistical significance.

## Introduction & background

The majority of patients with unstable spinal fractures will have metal fixation hardware implanted in their spine, including rods, screws, plates, wires, cages, and hooks, resulting in severely degraded magnetic resonance (MR) quality due to local susceptibility changes. These artifacts are mainly caused by local field inhomogeneities resulting from spin-resonance differences between the metal and surrounding soft-tissue, altering both phase and frequency of the MRI signals and scales with magnetic field strength [1–3]. The result is signal loss within the implants and the nearby tissues, distortion of the metallic implant, localized geometric distortion, and areas of high signal intensity (pile-up) around the metallic implants. As mentioned above, the resulting image degradation limits the diagnostic information available from these MR images.

### Reducing metal artifacts in MRI

Several techniques exist to reduce distortions when imaging near metal implants. One technique is pre-polarization, which relies on a pre-polarizing pulse and a subsequent readout field that is much lower in strength than conventional MR static fields. Pre-polarization has been shown to be effective in reducing metal artifacts as compared with conventional MRI, but implementation requires specialized hardware [2]. This makes the technique less than ideal, as it cannot be implemented easily on the existing installed base of MR scanners. Multi-Acquisition with Variable Resonances Image Combination (MAVRIC) [4] and iterative decomposition of water and fat with echo asymmetry and least-squares estimation (IDEAL) [5, 6] have also been introduced and used. However, the broad spectral coverage inherent in the MAVRIC technique may introduce magnetization transfer effects and/or develop alternative contrast between chemical species (e.g., water and fat). Furthermore, the total imaging times of these sequences can be on the order of 15 to 20 min, thereby rendering them impractical for clinical use.

View-angle-tilting (VAT) is another technique that can be used to correct metal artifacts. VAT uses a compensation gradient equal in amplitude to the slice-selection gradient, at an orientation that tilts the viewing angle and corrects for shifts resulting from field inhomogeneity. This technique has been implemented in structural imaging as the metal artifact reduction sequence (MARS) and has been shown to effectively reduce distortions, albeit at some cost, to SNR [5]. An addition to VAT, which includes additional z-phase encoding, has recently been developed and has shown promise in imaging near metal implants [7]. This technique, Slice Encoding for Metal Artifact Reduction (SEMAC) [7, 8], is extremely effective in reducing in-plane and through-plane artifacts, including signal pile-up, and is capable of acquiring structural images in a clinically feasible amount of time [3, 9].

This technique (VAT/SEMAC) has shown the most promise and is the current method of choice for metal suppression in centers and scanners where the technique is available. SEMAC is implemented on commercial scanners under various names such as WARP (Siemens Healthcare) and MAVRIC-SL [4, 10] (GE Healthcare). Although all these metal artifact-reducing MR pulse sequences have proven useful for orthopedic applications [4, 10–13], it has been challenging to apply these methods for better visualization and detection of normal and pathologic tissues in patients with metal implants. This is largely due to the induced susceptibility of artifacts resulting from the large amount of hardware typically used in unstable spine injuries. A spine study, presented at the 2013 joint spine section meeting, investigated the postoperative MRI artifacts with cobalt-chromium versus titanium spinal instrumentation [14]. However, this study was conducted on a 1.5T scanner and did not use any metal suppression techniques. Another study showed that SEMAC-SPIR MRI was superior to the other types of fat-suppressed MRI, such as SEMAC-IR or T_2_-weighted STIR MRI in spine transpedicular spondylodesis patients on 3T scanner [15].

To the best of our knowledge, systematic characterization of these artifacts in the presence of various types of spinal instrumentation has not yet been performed on high field scanners (3T). A thorough study of this will allow us to develop and establish comprehensive guidelines for reliably imaging the spinal cord in the presence of metal implantations. It is, therefore, the aim of this work to test the current MRI pulse sequences, which are increasingly becoming available in the modern MR systems, on the spinal cord to assess its effectiveness in visualizing the spinal cord in the presence of the metallic hardware currently being used in clinical SCI management.

## Methods & materials

**Fig. 1:**
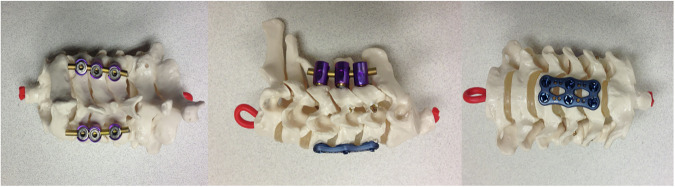
Cervical spine model with surgical stabilization hardware, anterior, posterior, and side view.

As a first step, we tested and optimized metal-suppression MR pulse sequences in various commonly used spinal implants using in vitro phantom models. This was done by developing realistic agar-suspended spine phantom models with the various metal implants currently being used to stabilize the spine and by evaluating the effects of MR-introduced distortion in the spinal cord. Spine model phantoms of various configurations were initially constructed to include several forms of instrumentation. Each spine model was assembled and instrumented with stabilization hardware, including anterior fixation plates, posterior rods with pedicle screws, and Kirschner wire in several combinations. All the hardware was installed by trained neurosurgeons in the same configurations as would be used in spinal surgery. A total of six models were implanted: cervical-spine with anterior plate and rods/screws (Fig. 1), cervical-spine with anterior plate, cervical-spine with rods/screws, cervical-spine with Kirschner wire, thoracic-spine with rods/screws, and thoracic-spine with anterior plate and rods/screws. After instrumentation, each model was suspended in a 2% agar gel using a stalk of asparagus as a spinal cord surrogate [16], which provided good contrast with the agar gel in both T1- and T2-weighted MR images (Fig. 1 in supplementary material).

The phantoms were then imaged on a 3T scanner using the Philips orthopedic metal artifact reduction sequence (O-MAR) sequence. The O-MAR sequence allows for VAT and three pre-defined levels of SEMAC encoding: weak (9-step), moderate (17-step), and strong (25-step), with step number referring to the amount of additional z-phase encoding. Phantoms were scanned using typical clinical T1- and T2-weighted fast spin-echo (FSE) sequences in sagittal and axial orientations to establish a baseline for metal artifact. Bandwidth was maximized for the initial base sequence to reduce magnetic susceptibility artifact, and O-MAR sequences were optimized for the shortest possible scan times given levels of metal suppression. To maintain shorter imaging times, half-scan (partial Fourier) factors were used.

After imaging, the spinal cord surrogate was segmented for each phantom and contrast/orientation using ITK-SNAP software. Segmentation was performed on the image with maximum suppression to provide the most artifact-free image possible. The segmentation mask was then overlaid to all images for each sequence (FSE, weak SEMAC, moderate SEMAC, and strong SEMAC) to allow for quantification of image characteristics for the spinal cord. A decrease in coefficient of variance (CoV) in the spinal cord region signal was used as a preliminary marker for reduction of artifact resulting from susceptibility artifact/intensity pile-up. Quantitative analysis was performed using in-house developed code written in Python.

### Subject recruitment

Sixteen adult subjects with SCI were recruited for this study. Female subjects (*n* = 8) ranged in age from 27 to 81 years, with a mean age of 63.4, and male subjects (*n* = 8) ranged from 38 to 75 years with a mean age of 56.0. **Inclusion Criteria:** Subjects age 18 years of age or older with a SCI of any severity (AIS A-D) along the cervical or thoracic spinal cord were included if they had any of the following spinal instrumentation configurations: titanium Kirschner wire (K-wire) at any cervical levels; a cervical anterior plate and screw system; a posterior cervical fusion with spinal rods and screws across a maximum of eight vertebral levels, with or without an anterior plate and screw system; and a posterior thoracic fusion with spinal rods and screws across a maximum of six vertebral levels. **Exclusion Criteria:** Subjects were excluded if they are unable to tolerate MRI without sedation; have an underlying neuromuscular, chromosomal or genetic diagnosis; have a diagnosis of infantile, juvenile or adolescent idiopathic scoliosis; have medical problems that interfere with lying supine for 60 min; have a suspected conversion syndrome; have a baclofin pump; pregnant; have cardiac pacing or cardiac implants; have full spinal instrumentation with complete circuits along the full length of the spinal column; have spinal instrumentation made of stainless steel; have implantable functional electrical stimulation systems in the chest or pelvic region; have spinal stimulation system; have uncontrolled autonomic dysreflexia; are dependent upon mechanical ventilation; have a tracheostomy tube containing metal; or; are unable or unwilling to consent to participate.

### Data analysis

Four board-certified neuroradiologists performed a qualitative analysis of the patient scans, both with and without metal suppression, and ranked them independently based on the criteria shown below. All reviewers were blinded to the MRI data and evaluated and graded the degree of the extent of the artifact as it involved obstruction of the central canal. A detailed scoring of the images with and without O-MAR was performed by the reviewers to evaluate the images for conspicuity of the neural foramen, mitigation of artifact, visualization of the spinal cord, and homogeneity of CSF (Table 1). These five rating criteria were established after discussions with four board certified neuroradiologist. According to neuroradiologists, these factors are the most important for radiological characterization of the spinal cord in their practices. These criteria were used to assess image degradation due to motion artifacts, susceptibility artifacts, and non-homogeneity artifacts. Rater scores were recorded to represent the following statements:

- The suppressed image being markedly better than the unsuppressed image
- The suppressed image being mildly better than the unsuppressed image
- No preference
- The unsuppressed image being mildly better than the suppressed image
- The unsuppressed image being markedly better than the suppressed image

**Table 1:** Criteria for the visual evaluation of images pre and post metal reduction scans.

Categories	Rating Scale used for each category
**Conspicuity of neural foramen**	1- The suppressed image being markedly better than the unsuppressed image
**Mitigation of Artifact**	2-The suppressed image being mildly better than the unsuppressed image
**Visualization of spinal cord**	3- No preference
**Homogeneity of CSF**	4- The unsuppressed image being mildly better than the suppressed image
	5- The unsuppressed image being markedly better than the suppressed image

Scores were summarized by rater for each outcome using means and standard deviations. Agreement among rater scores was evaluated using Gwet’s AC2 statistic [17]. Rater scores were modeled using linear regression in the context of generalized estimating equations to account for clustering among measurements from the same rater and to provide robust standard errors and confidence intervals.

### Pulse sequence parameters

All subjects were scanned on a Philips 3T Ingenia. The imaging parameters for various sequences are outlined in Table 1 in the supplemental material. The scanning protocol was uniform across both the cervical and thoracic regions of the spinal cord.

### Phantom results

**Fig. 2:**
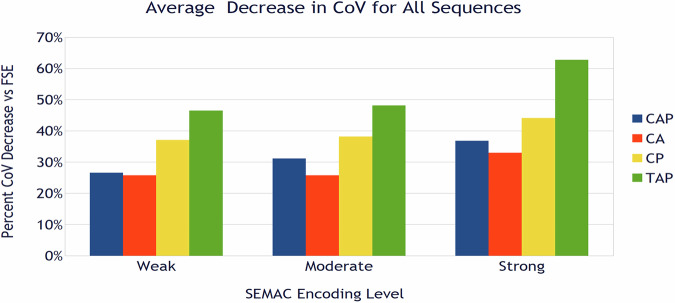
Decrease in CoV compared with the convention FSE sequence averaged for all contrasts and orientations acquired with the O-MAR sequence by SEMAC encoding level for phantoms: Cervical posterior rods/screws with anterior plate (CAP), Cervical anterior plate/screw (CA), Cervical posterior rods with pedicle screws (CP), and Thoracic posterior rods/screws with anterior plate (TAP).

**Fig. 3:**
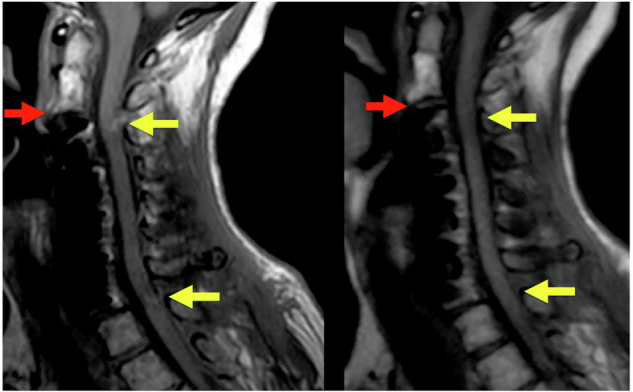
In-vivo images of a volunteer with spinal cord instrumentation using conventional (left) and O-MAR sequence (right). Pile-up artifact is visible present in the spinal cord midline in the conventional image (yellow arrows), susceptibility artifact obscures the C2-C3 intervertebral disc (red arrows).

The O-MAR sequence showed reduction of metal artifacts and provided a clearer view of the cord (Fig. 2, supplementary material). The MRI acquisition times for the SEMAC sequence with 0, 9, 17, and 25 steps were 2 min 23 s, 3 min 7 s, 7 min 18 s, and 9 min 23 s, respectively. Average improvements of 34, 36, and 44% in co-variance (CoV) were found for weak, moderate, and strong suppression, respectively, for all phantoms. Greater SEMAC encoding provided greatest change for axial images, with average improvements of 25, 29, and 43%, while sagittal images remained comparable at 42, 42, and 46%. Figure 2 and 3 shows the average decrease in CoV for the spinal cord, averaged for all sequences for each phantom and level of SEMAC encoding. Axial sequences showed a greater decrease in CoV with increased scan time as compared with sagittal sequences (Fig. 3 supplementary material).

### Human results

The phantom-tested sequences were subsequently used to guide metal suppression strategies in SCI participants. In-vivo scans were successful in all 16 subjects. In general, participants tolerated the scanning and were able to provide motion-free data in most cases. Overall examination of in-vivo images shows effective suppression of metal artifact. In general, as seen in the phantoms, weak (9-step) SEMAC suppression was effective at removing through-plane pile-up artifact and providing clear, unobscured views of the cervical spinal cord in the presence of anterior and posterior hardware, including rods, screws, and plates. Images obtained with the O-MAR sequence show decreased artifact compared with conventional clinical sequences and could be acquired with acceptable scan times and SAR levels. Figures 3 and 4 in supplementary material show preliminary data illustrating reduction in metal artifact. Table 2 shows the raw mean scores and standard deviations by rater for each measure. Table 2 in the supplementary material quantifies the agreement among raters using the Gwet AC2 statistics. The closer the AC2 is to 0, the less agreement there is, and the closer AC2 is to 1, the more agreement there is. AC2 was calculated to assess agreement among all raters and among all raters excluding rater #2. The agreement between the raters was moderate (0.41 to 0.6) to substantial (0.61 to 0.8) for most measures, although some were in the fair range (0.21 to 0.4).

**Table 2:** Summary statistics for each outcome by rater.

Outcome	Rater#1 (mean, std)	Rater#2 (mean, std)	Rater#3 (mean, std)	Rater#4 (mean, std)
Conspicuity of Neural Foramen				
T1-weighted-axial	3.08 (1.24)	2.92 (0.90)	3.00 (1.13)	2.83 (1.03)
T1-weighted-sagittal	3.00 (0.35)	2.82 (0.88)	3.00 (0.61)	2.76 (0.75)
T2-weighted-axial	3.07 (0.27)	2.71 (1.07)	2.86 (0.53)	2.93 (0.73)
T2-weighted-sagittal	3.06 (0.25)	2.94 (0.68)	3.06 (0.44)	3.13 (0.62)
Mitigation of Artifact				
T1-weighted-axial	2.92 (1.00)	2.83 (0.83)	3.75 (0.75)	2.75 (0.87)
T1-weighted-sagittal	3.06 (1.09)	2.94 (1.34)	2.76 (1.35)	2.47 (1.66)
T2-weighted-axial	3.00 (1.04)	3.07 (0.73)	2.93 (0.73)	3.29 (0.73)
T2-weighted-sagittal	3.13 (0.89)	3.00 (0.97)	2.63 (0.89)	2.82 (1.11)
Visualization of Spinal Cord				
T1-weighted-axial	2.92 (1.24)	2.92 (1.00)	3.08 (0.90)	2.67 (0.98)
T1-weighted-sagittal	2.59 (1.18)	3.00 (1.32)	3.12 (1.45)	2.47 (1.46)
T2-weighted-axial	3.79 (1.25)	3.14 (0.86)	2.71 (1.07)	2.79 (0.80)
T2-weighted-sagittal	3.38 (1.26)	3.13 (0.96)	2.75 (0.93)	2.88 (0.96)
Homogeneity of CSF				
T1-weighted-axial	3.08 (1.16)	2.91 (0.90)	2.67 (1.07)	2.83 (1.19)
T1-weighted-sagittal	2.94 (1.30)	3.12 (1.11)	2.41 (1.23)	2.76 (1.35)
T2-weighted-axial	3.43 (1.60)	3.07 (1.07)	3.21 (1.47)	3.07 (1.14)
T2-weighted-sagittal	3.44 (1.32)	3.50 (0.96)	3.13 (1.15)	3.44 (0.89)

Table 3 shows the model-estimated means and their 95% confidence intervals across raters and implant types. All of the confidence intervals for means in Table 2 of the supplemental material include a value of 3, which shows that, on average, neither image type was preferred. Table 4 shows how the mean score for each image type varies by implant type, as well as the difference in mean score between the two implant types. For most outcomes, the confidence intervals for the mean difference included 0, so neither posterior rod nor anterior plate was favored for suppressed or unsuppressed images. The exceptions to this were the T2 weighted-axial image for Visualization of Spinal Cord, the T1 weighted-axial image for Homogeneity of CSF, and the T2 weighted-axial image for Homogeneity of CSF. In these three cases, the confidence intervals included only positive numbers, so the images for the anterior plate favored the suppressed image relative to the images from the posterior rod. For most image types, neither the posterior rod nor anterior plate scores were significantly different from a neutral value of 3. The exceptions were T2 weighted-axial images for conspicuity of neural foramen, visualization of spinal cord, and homogeneity of CSF, and the T1 weighted-axial image for homogeneity of CSF in the anterior plate. In all of these situations, the upper bound of the confidence interval was below 3, so the suppressed image was favored.

**Table 3:** Model-estimated mean score for each outcome.

Outcome	Mean Score (95% CI) unadjusted
Conspicuity of Neural Foramen	
T1-weighted-axial	2.96 (2.46–3.46)
T1-weighted-sagittal	2.90 (2.74–3.05)
T2-weighted-axial	2.89 (2.69–3.10)
T2-weighted-sagittal	3.05 (2.91–3.18)
Mitigation of Artifact	
T1-weighted-axial	3.06 (2.90–3.23)
T1-weighted-sagittal	2.81 (2.41–3.21)
T2-weighted-axial	3.07 (2.88–3.26)
T2-weighted-sagittal	2.89 (2.65–3.14)
Visualization of Spinal Cord	
T1-weighted-axial	2.90 (2.43–3.36)
T1-weighted-sagittal	2.79 (2.32–3.27)
T2-weighted-axial	3.11 (2.67–3.54)
T2-weighted-sagittal	3.03 (2.67–3.39)
Homogeneity of CSF	
T1-weighted-axial	2.88 (2.37–3.38)
T1-weighted-sagittal	2.81 (2.41–3.21)
T2-weighted-axial	3.20 (2.57–3.82)
T2-weighted-sagittal	3.38 (2.96–3.79)

**Table 4:** Model-estimated mean score for each outcome by implant type.

Outcome	Posterior Rod (95% CI)	Anterior Plate (95% CI)	Difference (95% CI)
**Conspicuity of Neural Foramen**			
T1-weighted-axial	3.08 (2.54–3.62)	2.13 (0.91–3.33)	0.96 (−0.37–2.29)
T1-weighted-sagittal	2.85 (2.71–3.00)	2.92 (2.33–3.50)	−0.06 (−0.66–0.54)
T2-weighted-axial	2.90 (2.64–3.16)	**2.63** (**2.45**–**2.80)**	0.28 (−0.03–0.59)
T2-weighted-sagittal	3.02 (2.86–3.18)	3.17 (2.89–3.43)	−0.14 (−0.46–0.17)
**Mitigation of Artifact**			
T1-weighted-axial	3.06 (2.87–3.24)	3.13 (2.61–3.64)	−0.07 (−0.62–0.48)
T1-weighted-sagittal	2.81 (2.33–3.29)	2.83 (2.02–3.65)	−0.02 (−0.96–0.92)
T2-weighted-axial	3.05 (2.80–3.30)	3.25 (2.90–3.60)	−0.20 (−0.63–0.23)
T2-weighted-sagittal	2.84 (2.54–3.15)	3.25 (2.79–3.71)	−0.41 (−0.96–0.14)
**Visualization of Spinal Cord**			
T1-weighted-axial	2.97 (2.50–3.44)	2.13 (0.91–3.34)	0.85 (−0.45–2.15)
T1-weighted-sagittal	2.75 (2.22–3.28)	2.33 (1.06–3.61)	0.42 (−0.96–1.80)
T2-weighted-axial	3.30 (2.80–3.80)	**2.25** (**1.90**–**2.60)**	**1.05** (**0.44**–**1.66)**
T2-weighted-sagittal	3.11 (2.64–3.59)	2.92 (2.33–3.50)	0.20 (−0.56–0.95)
**Homogeneity of CSF**			
T1-weighted-axial	3.00 (2.47–3.53)	**1.88** (**1.00**–**2.74)**	**1.13** (**0.11**–**2.14)**
T1-weighted-sagittal	2.83 (2.37–3.30)	2.67 (1.40–3.85)	0.17 (−1.11–1.44)
T2-weighted-axial	3.43 (2.68–4.17)	**2.25** (**1.90**–**2.59)**	**1.18** (**0.36**–**2.00)**
T2-weighted-sagittal	3.36 (2.82–3.91)	3.25 (2.85–3.65)	0.11 (−0.56–0.78)

## Discussion

Management and treatment of acute traumatic unstable spine injury often require the implantation of metallic stabilization hardware (instrumentation), which includes rods, hooks, braided cables, plates, screws, and interbody cages to provide immediate treatment for spinal instability. In addition to optimizing neurological recovery, instrumentation realigns the spinal column, creates a condition in which fractures can heal, and facilitates engagement in physical and occupational therapy, unencumbered by external orthoses (for example, halo vest and back brace) and fracture precaution protocols. The instrumentation is typically composed of titanium and cobalt-chromium alloys. While these metallic structures provide obvious benefits in the early management of acute traumatic SCI, they also cause artifacts with MR imaging and unfortunately hamper the visualization of neurological structures. These artifacts, called susceptibility artifacts, occur primarily due to differences in magnetic susceptibility between two adjacent materials that are imaged, and is field strength dependent (e.g., spine tissue and metal). The higher the MR field strength the higher the susceptibility induced artifacts.

The paradox of surgical stabilization and instrumentation of the spine is the resultant degradation in the quality of subsequently performed imaging studies. As neurological deterioration can occur after surgical stabilization, imaging is principally relied upon to determine the root cause. Therefore, artifact mitigation can improve assessment of the neural elements after surgery. The limited visualization of the spinal cord parenchyma following instrumentation is an important cause of screening failure when enrolling potential patients into promising clinical trials that use cell-mediated therapies. Given the already existing challenges of recruitment for SCI clinical trials [18], these methods provide a means to successfully image the instrumented spine. Mitigation of MRI artifacts caused by metallic implants currently used in spine trauma management will allow for improved human spinal cord imaging near metal implants. There have been recent developments in the field of metal suppression using MR pulse sequences, but most have focused on studying implants in the musculoskeletal system and not systematically in the spine implants [11–13].

The results demonstrate the potential of advanced MRI techniques on a high field 3T scanner, specifically the O-MAR sequence and varying SEMAC encoding strategies, in significantly reducing metal artifacts that typically obscure spinal cord visualization in patients with metallic implants. Our results indicate that weak SEMAC encoding provides an effective balance between image quality and scan time, allowing for clear, artifact-free views of the cervical spinal cord. While stronger SEMAC encoding offers further improvements in image clarity, these gains may not justify the extended scan times required for clinical use, especially in the cervical region. In contrast, for thoracic regions with larger instrumentation, longer scan times may be more appropriate to optimize visualization. In general, SEMAC also offers the benefit of increased SNR due to the additional z-phase encoding steps acting as additional signal averages. Overall, however, improvements in image quality are not likely to justify the severely extended scan times required for strong or even moderate SEMAC encoding. At the cervical level, weak SEMAC encoding was effective in providing clear, unobstructed views of the spinal cord without pile-up artifact at the midline in sagittal images or at the cord boundaries in axial images. Due to larger instrumentation in the thoracic spine, longer scans with stronger SEMAC encoding may be appropriate to provide the best possible view of the spinal cord. In all, substantive improvements in metal artifact reduction resulting in unobstructed views of the spinal cord in the phantoms studied can be achieved with increases of approximately 0.5–3.5 min of additional scan time per scan. For a full clinical cervical protocol, this translates to 4–8 min of added scan time to provide useful diagnostic images of the spinal cord in the presence of metal implants, where conventional imaging would be unable to yield usable images. Our phantom study results showed good mitigation of metal artifact, particularly in cervical instrumentation, with acceptable SAR levels and scan times.

Clinically, it is more common to image subjects with spine stabilization implants at lower field strengths (1.5T or lower), as susceptibility artifact scales with field strength. However, using modern protocols with VAT/SEMAC techniques, it is possible to take advantage of higher resolution and improved signal from high-field 3T magnets. Several studies have shown relationships between spinal cord injury/recovery and morphologic features such as cross-sectional area, atrophy, gray matter/white matter volume, and tissue bridge characteristics [19–22]. The ability to image the cord in the presence of implants at higher fields can provide higher quality data for follow up and longitudinal examination of the injured spinal cord.

Optimizing metal-suppression sequences for specific applications is a time-intensive process requiring both phantom and human subject imaging, as well as familiarity with sequence parameters. As these advanced metal suppression techniques become more available, many institutions may not have the personnel or resources necessary to implement them and, as a result, will be unable to fully exploit state-of-the-art metal suppression techniques for spinal cord imaging. As with most imaging studies, the translation of results from phantom experiments does not necessarily replicate in humans using the same optimized techniques. Factors such as patient motion, tissue susceptibility differences, eddy current effects in human tissues, and partial volume effects at tissue interfaces can all contribute to discrepancies between phantom and in vivo results. Furthermore, in clinical practice, radiologists prioritize the visibility of key anatomical structures or pathological findings once these regions are clearly seen, further reducing minor residual artifacts rarely improves subjectively rated image quality or diagnostic confidence.

The optimization process described in this paper will greatly help the imaging community, both in imaging patients with SCI and in clinical trials, to obtain images of the spinal cord with good reproducibility and reliability in the presence of metal hardware. The significance of this work lies in achieving a better understanding of the local environment after SCI and the impact of treatments on the spinal cord, leading to improved longitudinal management, whether patients are involved in routine clinical care, neurorehabilitation, or experimental therapeutic trials.

This study has a few limitations. The first and foremost is the generalizability of these image acquisition parameters and analyses to all other spinal diseases that involve metallic implants besides SCI. One can adopt these findings but should carefully adjust the MR parameters to achieve maximum suppression of the metal artifacts. Also, it may be interesting to have the orthopedic surgeon and neurosurgeon grade the MRI and see if they have a preference. In this study, we focused exclusively on titanium, as it remains the most widely used material for spine surgery instrumentation. However future studies should consider choice of instrumentation and patient-specific factors, surgical goals as well as clinical correlations. Another limitation is that relatively small sample size. This study was designed as an initial proof-of-concept evaluation to determine whether the metal-suppression MR pulse sequence meaningfully improves visual assessment across key qualitative features, including neural foraminal conspicuity, artifact mitigation, spinal cord visualization, and CSF homogeneity. Although the sample size does not allow robust statistical power or detection of more subtle differences, the qualitative improvements observed were consistent and encouraging. These findings support the feasibility and potential clinical value of applying metal-suppression techniques in postoperative spine imaging. We view this work as hypothesis-generating and expect that validation in larger cohorts will be essential to confirm and quantify these effects more rigorously. Another limitation in this study is the assumption that improved SNR resulting from the use of 3T imaging is always superior to 1.5T in clinical application provided that the metal artifact impact is mitigated. Ideally, a comparison to 1.5T in terms of the image preference metrics would provide a more complete picture of the benefits/trade-offs, but the inclusion of an additional scanner and scan session was not feasible for the scope of this study. In the spinal cord pathology cohort used in this study, CoV may not be a reliable measure of image quality due to injury-induced inhomogeneity within the cord. In contrast, our phantom is relatively homogeneous and easily quantified. Hence, we chose not to perform the CoV analysis in the patient population.

The last few decades have seen a significant increase in the use of MRI methods in SCI patients. These techniques vary from anatomical localization using various contrast generating structural MRI scans, to diffusion weighted MRI to look at the white matter structures to name a few. Many of these methods are proving to be helpful for localization of the SCI injury, provide quantitative spinal cord cross sectional measurements of the lesions, and sparred tissue bridges, and non-invasively show white matter disruption in SCI patients. Numerous studies have shown that these biomarkers obtained from MRI could be used for longitudinal follow up of patients during clinical trials, non-invasively observing the injury site and beyond during neurorehabilitation and to stratification of patients for various therapies. Thus, reliable visualization and quantification of spinal cord features via MRI is essential for clinical decision-making and research in SCI.

In conclusion, this work underscores the critical role of refined imaging techniques in enhancing the clinical assessment of spinal cord injuries in the context of modern surgical hardware. Based on the results of these phantom and in-vivo experiments, cervical spinal cord imaging can be performed effectively at 3T even with a small amount of SEMAC encoding (9 steps). This can be done with only a minimal increase in scan time while gaining the potential benefits of increased resolution and SNR at high field. Maximizing bandwidth and enabling VAT/SEMAC with this encoding strength is achievable with most modern 3T MR scanners, irrespective of manufacturer. Further development and integration of these techniques into routine clinical practice could have profound implications, not only for the management of SCI but also for the advancement of research and therapeutic interventions in this field.

## Supplementary information

Supplemental Material

## Data Availability

Data provided upon request.
