# Supplementary material for: MRI metal artifact characterization in patients with spinal cord injury at 3 Tesla
Source: Spinal Cord. 2026 May 29;64(7):654–60. doi: 10.1038/s41393-026-01222-0 (PMC13345889; doi:10.1038/s41393-026-01222-0)
Supplement: Supplementary file 1 — Supplemental Material [file 41393_2026_1222_MOESM1_ESM.docx]

| **T1 Weighted Sagittal** | **Base** | **SEMAC weak** | **SEMAC moderate** | **SEMAC strong** |
| --- | --- | --- | --- | --- |
| TE (ms) | 5.2 | 16.9 | 10.4 | 10.8 |
| TR (ms) | 349 | 545 | 620 | 529 |
| Pixel BW (Hz) | 869 | 869 | 869 | 869 |
| SEMAC steps | none | 9 | 17 | 25 |
| Voxel size (mm) | 0.42 x 0.42 | 0.42 x 0.42 | 0.42 x 0.42 | 0.42 x 0.42 |
| Slice Thickness (mm) | 3 | 3 | 3 | 3 |
| Slices | 15 | 15 | 19 | 25 |
| TA (s) | 143 | 187 | 438 | 563 |
| Partial Fourier | none | 0.6 | 0.6 | 0.525 |
|  |  |  |  |  |
| **T1 Weighted Axial** | **Base** | **SEMAC weak** | **SEMAC moderate** | **SEMAC strong** |
| TE (ms) | 5.6 | 17.3 | 11.2 | 11.2 |
| TR (ms) | 439 | 613 | 620 | 529 |
| Pixel BW (Hz) | 880 | 880 | 880 | 880 |
| SEMAC steps | none | 9 | 17 | 25 |
| Voxel size (mm) | 0.39 x 0.39 | 0.39 x 0.39 | 0.39 x 0.39 | 0.39 x 0.39 |
| Slice Thickness (mm) | 4 | 4 | 4 | 4 |
| Slices | 17 | 17 | 17 | 25 |
| TA (s) | 144 | 174 | 360 | 532 |
| Partial Fourier | none | 0.6 | 0.6 | 0.525 |
|  |  |  |  |  |
| **T2 Weighted Sagittal** | **Base** | **SEMAC weak** | **SEMAC moderate** | **SEMAC strong** |
| TE (ms) | 100 | 89 | 80 | 60 |
| TR (ms) | 3942 | 2100 | 2665 | 2333 |
| Pixel BW (Hz) | 1064 | 1062 | 1062 | 1062 |
| SEMAC steps | none | 9 | 17 | 25 |
| Voxel size (mm) | 0.42 x 0.42 | 0.42 x 0.42 | 0.42 x 0.42 | 0.37 x 0.37 |
| Slice Thickness (mm) | 3 | 3 | 3 | 3 |
| Slices | 15 | 15 | 17 | 25 |
| TA (s) | 150 | 334 | 496 | 747 |
| Partial Fourier | none | 0.775 | 0.675 | 0.6 |
|  |  |  |  |  |
| **T2 Weighted Axial** | **Base** | **SEMAC weak** | **SEMAC moderate** | **SEMAC strong** |
| TE (ms) | 100 | 107 | 80 | 60 |
| TR (ms) | 4435 | 2100 | 2665 | 2838 |
| Pixel BW (Hz) | 880 | 880 | 880 | 880 |
| SEMAC steps | none | 9 | 17 | 25 |
| Voxel size (mm) | 0.39 x 0.39 | 0.39 x 0.39 | 0.39 x 0.39 | 0.39 x 0.39 |
| Slice Thickness (mm) | 4 | 4 | 4 | 4 |
| Slices | 17 | 17 | 17 | 25 |
| TA (s) | 195 | 346 | 405 | 630 |
| Partial Fourier | none | 0.775 | 0.675 | 0.6 |

**Table 1. Spine Imaging Acquisition Parameters.**

| **Outcome** | **AC2** |
| --- | --- |
| Conspicuity of Neural Foramen |  |
| T1-weighted-axial | 0.635 |
| T1-weighted-sagittal | 0.644 |
| T2-weighted-axial | 0.727 |
| T2-weighted-sagittal | 0.637 |
| Mitigation of Artifact |  |
| T1-weighted-axial | 0.404 |
| T1-weighted-sagittal | 0.210 |
| T2-weighted-axial | 0.581 |
| T2-weighted-sagittal | 0.466 |
| Visualization of Spinal Cord |  |
| T1-weighted-axial | 0.627 |
| T1-weighted-sagittal | 0.383 |
| T2-weighted-axial | 0.530 |
| T2-weighted-sagittal | 0.517 |
| Homogeneity of CSF |  |
| T1-weighted-axial | 0.645 |
| T1-weighted-sagittal | 0.347 |
| T2-weighted-axial | 0.690 |
| T2-weighted-sagittal | 0.574 |

**Table 2. Agreement amongst all the raters.**

**Figure 1** – 3D reconstruction of a non-instrumented model showing the surrogate cord. The image shown is a surface rendering from data acquired using a high resolution T1 weighted MR scan on the 3T scanner.

**Figure 2** – Cervical phantom with anterior plate and rods/screws imaged with conventional FSE sequence (left) and time/quality optimized O-MAR sequence (right) for T1 and T2 contrasts with a weak suppression scheme. Signal pile-up on the cord midline (yellow arrows) is not present in the optimized sequences.


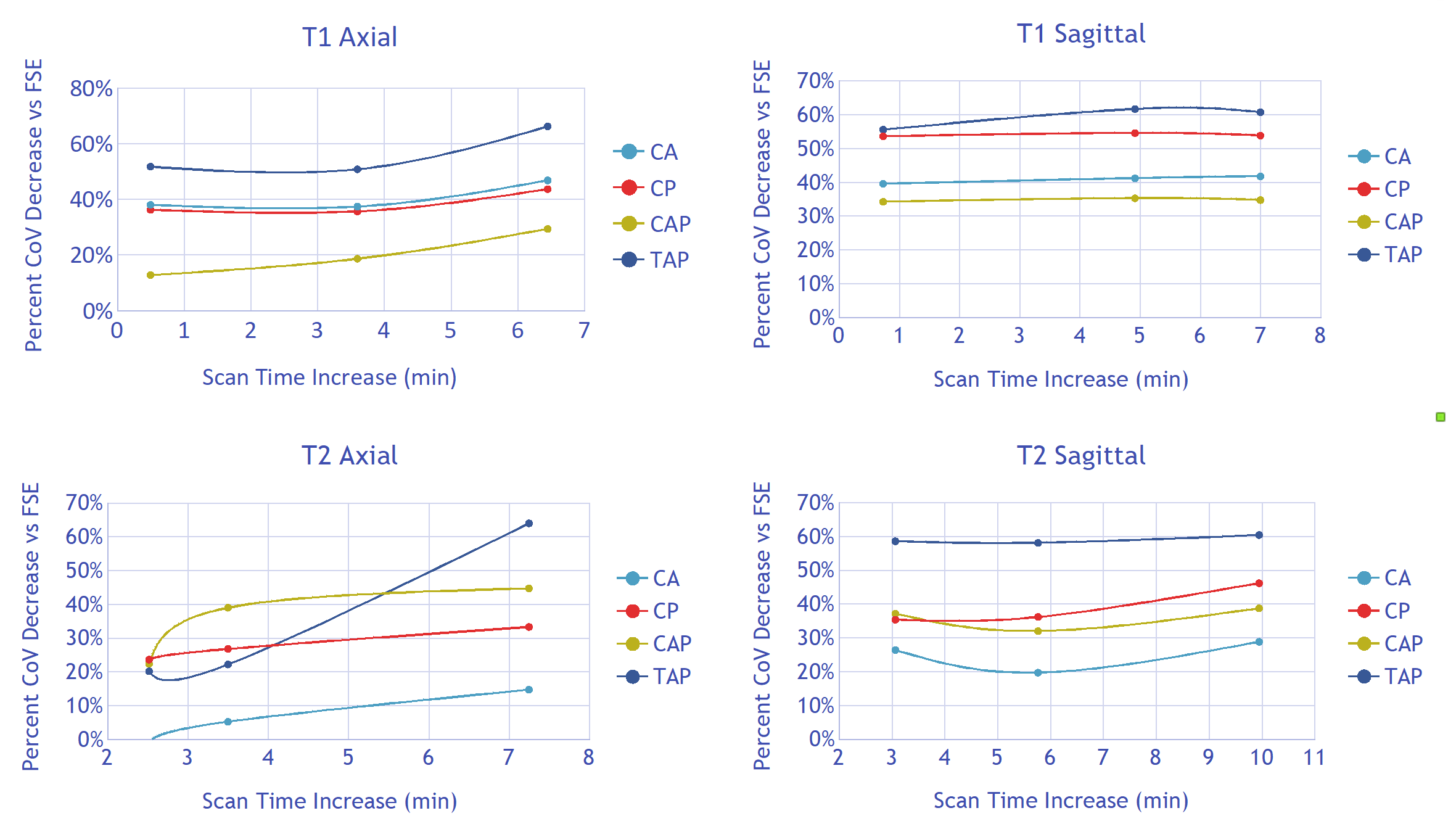


**Figure 3** – Percentage decrease in coefficient of variance from FSE versus scan time increase for all sequences and phantoms: Cervical posterior rods/screws with anterior plate (CAP), Cervical anterior plate/screw (CA), Cervical posterior rods with pedicle screws (CP), and Thoracic posterior rods/screws with anterior plate (TAP)

**Figure 4** – In-vivo images of a participant with an anterior plate/screws instrumentation using conventional (left) and O-MAR sequence (right). Metal artifact impact is minimal on the cord as predicted, but visibility of the vertebral bodies and discs (yellow arrow) is improved with metal suppression techniques (right).
